# Improvements in Patient Acceptance by Hospitals Following the Introduction of a Smartphone App for the Emergency Medical Service System: A Population-Based Before-and-After Observational Study in Osaka City, Japan

**DOI:** 10.2196/mhealth.8296

**Published:** 2017-09-11

**Authors:** Yusuke Katayama, Tetsuhisa Kitamura, Kosuke Kiyohara, Taku Iwami, Takashi Kawamura, Junichi Izawa, Koichiro Gibo, Sho Komukai, Sumito Hayashida, Takeyuki Kiguchi, Mitsuo Ohnishi, Hiroshi Ogura, Takeshi Shimazu

**Affiliations:** ^1^ Department of Traumatology and Acute Critical Medicine Osaka University Graduate School of Medicine Suita Japan; ^2^ Division of Environmental Medicine and Population Sciences Department of Social and Environmental Medicine Osaka University Graduate School of Medicine Suita Japan; ^3^ Department of Public Health Tokyo Women's Medical University Tokyo Japan; ^4^ Kyoto University Health Sevices Kyoto Japan; ^5^ Intensive Care Unit Department of Anesthesiology The Jikei University School of Medicine Tokyo Japan; ^6^ Department of Emergency Medicine Okinawa Prefectural Chubu Hospital Uruma Japan; ^7^ Clinical Research Center Saga University Hospital Saga Japan; ^8^ Osaka Municipal Fire Department Osaka Japan

**Keywords:** emergency medicine, emergency medical services, mobile health, telemedicine, public health

## Abstract

**Background:**

Recently, the number of ambulance dispatches has been increasing in Japan, and it is therefore difficult for hospitals to accept emergency patients smoothly and appropriately because of the limited hospital capacity. To facilitate the process of requesting patient transport and hospital acceptance, an emergency information system using information technology (IT) has been built and introduced in various communities. However, its effectiveness has not been thoroughly revealed. We introduced a smartphone app system in 2013 that enables emergency medical service (EMS) personnel to share information among themselves regarding on-scene ambulances and the hospital situation.

**Objective:**

The aim of this study was to assess the effects of introducing this smartphone app on the EMS system in Osaka City, Japan.

**Methods:**

This retrospective study analyzed the population-based ambulance records of Osaka Municipal Fire Department. The study period was 6 years, from January 1, 2010 to December 31, 2015. We enrolled emergency patients for whom on-scene EMS personnel conducted hospital selection. The main endpoint was the difficulty experienced in gaining hospital acceptance at the scene. The definition of difficulty was making ≥5 phone calls by EMS personnel at the scene to hospitals until a decision to transport was determined. The smartphone app was introduced in January 2013, and we compared the patients treated from 2010 to 2012 (control group) with those treated from 2013 to 2015 (smartphone app group) using an interrupted time-series analysis to assess the effects of introducing this smartphone app.

**Results:**

A total of 600,526 emergency patients for whom EMS personnel selected hospitals were eligible for our analysis. There were 300,131 emergency patients in the control group (50.00%, 300,313/600,526) from 2010 to 2012 and 300,395 emergency patients in the smartphone app group (50.00%, 300,395/600,526) from 2013 to 2015. The rate of difficulty in hospital acceptance was 14.19% (42,585/300,131) in the control group and 10.93% (32,819/300,395) in the smartphone app group. No change over time in the number of difficulties in hospital acceptance was found before the introduction of the smartphone app (regression coefficient: −2.43, 95% CI −5.49 to 0.64), but after its introduction, the number of difficulties in hospital acceptance gradually decreased by month (regression coefficient: −11.61, 95% CI −14.57 to −8.65).

**Conclusions:**

Sharing information between an ambulance and a hospital by using the smartphone app at the scene was associated with decreased difficulty in obtaining hospital acceptance. Our app and findings may be worth considering in other areas of the world where emergency medical information systems with IT are needed.

## Introduction

In Japan, when emergency patients call for emergency medical service (EMS) at the scene, on-scene EMS personnel assess the patient’s condition and then transport the patient to a hospital that can accept and treat him or her [1]. In this process, ambulances can transport the patient to the hospital only after obtaining permission from the selected hospital via a phone call [1]; this permission is defined as hospital acceptance in Japan [2]. Recently, the number of emergency patients transported to a hospital by EMS has been increasing and exceeding the hospital capacity in Japan. Therefore, it is becoming more difficult to obtain permission and transport and accept emergency patients smoothly and appropriately, especially for severely ill patients and pregnant women [3]. Indeed, our previous study revealed that prehospital factors such as patient’s age and time of day were associated with difficulty in hospital acceptance at the scene by analyzing the ambulance records in Osaka City, Japan [4].

Digital information devices such as smartphones and tablet computers have been developing dramatically, and various medical information systems for EMS and medical institutions have also been introduced with the use of these devices in Japan [5]. If EMS personnel at the scene could see the real-time situation of patient transport and hospital acceptance by using a mobile app for smartphones and iPad, they would be able to transport emergency patients to the hospital more smoothly. However, it has not been sufficiently assessed whether the introduction of such information systems would improve the emergency patient transport process by an EMS.

Osaka City is the largest city in western Japan, and there are about 200,000 emergency dispatches every year. We developed a medical information system with smartphone app for an EMS system to facilitate hospital selection and the transport of emergency patients. We call this medical information system as ORION (Osaka emergency information Research Intelligent Operation Network system). It has been in operation in Osaka since January 2013. By analyzing the population-based ambulance records of the Osaka Municipal Fire Department (OMFD) before and after the introduction of ORION, this study aimed to assess the effects of the introduction of this medical information system for an EMS on the difficulty in obtaining hospital acceptance.

## Methods

### Study Design, Population, and Setting

This was a retrospective, population-based, observational study using ambulance records of the OMFD in Osaka City, Japan. The study period was 6 years, from January 1, 2010 to December 31, 2015. Among all emergency dispatches, this study enrolled emergency patients for whom EMS personnel at the scene selected the hospital, and it excluded those who were not transported or were transported to hospitals requested by the patients or their family and those who were transported between hospitals This study was approved by the ethics committees of Osaka University Graduate School of Medicine and Kyoto University Graduate School of Medicine. The ambulance records of the OMFD were considered administrative records, and the requirement of obtaining patients’ informed consent was waived. The researchers dealt only with anonymous data that were not linkable to the patients.

### EMS System and Hospitals in Osaka City

Osaka City, the largest metropolitan community in western Japan, had a population of about 2.7 million in 2017 and covers an area of 222 km^2^. The annual number of emergency patients transported by an EMS in Osaka City is about 200,000. The municipal EMS system is basically the same as that in the other areas of Osaka Prefecture, as previously described [6]. Briefly, an EMS system is operated exclusively by the OMFD and is activated by calling 119. The OMFD had 25 fire stations (60 ambulances) and one dispatch center in 2016. Usually, each ambulance has a crew of 3 emergency providers, including at least one emergency lifesaving technician who is a highly-trained prehospital emergency care provider authorized to use an automated external defibrillator to insert an intravenous line and administer adrenaline and to place advanced airway management [7]. Osaka City had 184 hospitals (32,645 beds) in 2015 [8]. Among those, 99 hospitals, including 6 critical care centers, were designated to accept life-threatening emergency patients from ambulances. During the study period, emergency dispatchers in Osaka City did not make phone calls to hospitals for acceptance; only ambulances crews at the scene selected appropriate hospitals, including critical care medical centers for the emergency patients.

### Smartphone App in the ORION System

EMS personnel at the scene operate a smartphone app connected to the ORION system for each emergency patient. When EMS personnel launch this app and register an emergency patient, the app screen for recording the prehospital time course of the patient’s transport is active (Multimedia Appendix 1). When EMS personnel touch the button labeled “arrival at the scene,” the time of arrival at the scene is recorded, and then the location is also recorded by activating the global positioning system on the smartphone. Next, when they leave the scene and touch the button labeled “departure from the scene,” the time is recorded and the status of the ambulance changes to “transporting to hospital.” On touching the button labeled “arrival at the hospital,” the arrival time is recorded, and the status changes to “during treatment of patient.” Ambulance statuses from the scene to the hospital are registered in the ORION cloud server and are also reflected on the screen of the medical institution list displayed on the app of other ambulances. For hospital selection, when an EMS personnel at the scene touches the button labeled “to patient check list” in the ambulance status screen, the app screen for recording the patient’s status such as vital signs and background becomes active (Multimedia Appendix 2). EMS personnel can choose symptoms displayed on the app screen that match the patient’s complaints, and then the appropriate treating hospitals are listed based on the patient’s condition. For example, Multimedia Appendix 3 shows the app screenshot for “chest pain,” on which EMS personnel can check items such as “ST-T change” and “dyspnea” for patients with chest pain. In Osaka, medical institutions are categorized based on the feasibility of treating these conditions [9]. When EMS personnel select the check items on this screen, this app shows the list of hospitals that can conduct treatment for potential etiology (or disease) such as emergency percutaneous catheter intervention. In the screen listing the medical institutions (Multimedia Appendix 4), EMS personnel can select the appropriate hospital for the emergency patient. Colored circles in the screen illustrate the status of emergency patients in each hospital as follows: A red circle means that another ambulance is transporting a patient to that hospital, a yellow circle means that medical staff are now treating a patient transported by another ambulance, and a blue circle means that the hospital is currently neither receiving nor treating any patient. When EMS personnel at the scene select an appropriate hospital for the patient, considering the status of the medical institutions displayed on the app screen, and touch the name of a medical institution, the hospital is automatically called. After receiving consent of hospital acceptance from the hospital, the on-scene EMS personnel start to transport the patient to the hospital. If the hospital rejects the request from EMS personnel, the number of telephone calls required until a receiving hospital is determined is also automatically totaled. EMS personnel at the scene can call critical care centers without filling in the patient’s checklist in the app if they judge that the patient is in critical condition.

**Figure 1:**
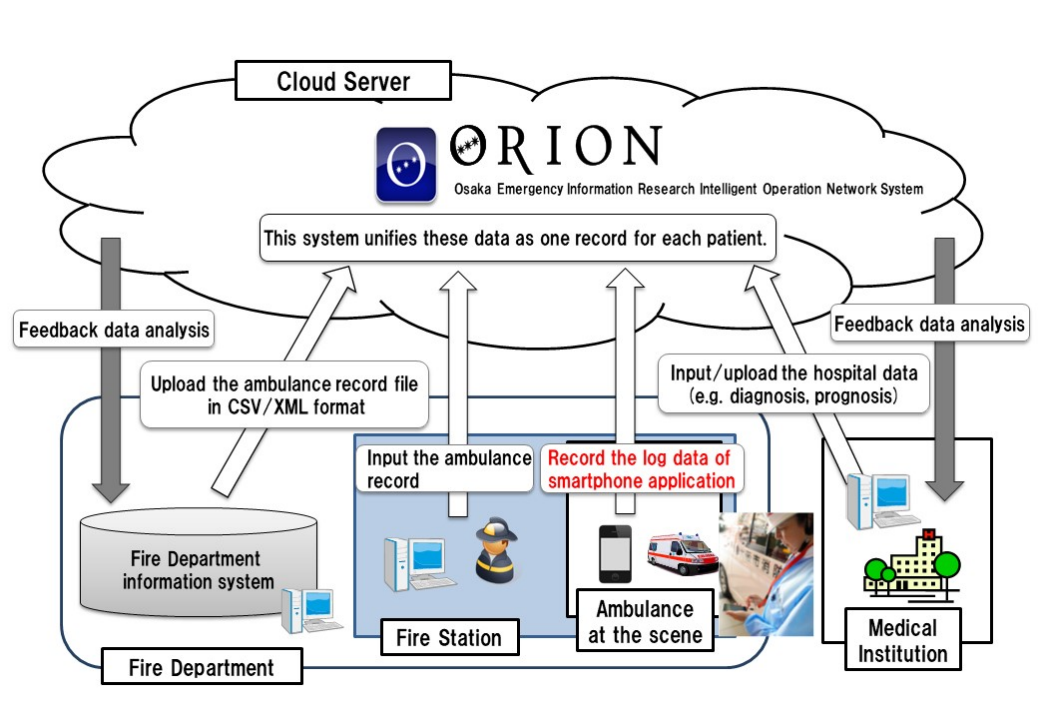
System configuration of Osaka emergency information Research Intelligent Operation Network system (ORION). All of the data consisting of smartphone app data, ambulance data, and hospital data are merged in the ORION cloud server and managed as one large database in Osaka.

The smartphone app data are accumulated in the ORION cloud server, and data managers in cooperation with dispatched EMS personnel directly input or upload the ambulance record of each emergency patient so that it can be merged with the app data. Furthermore, each hospital also directly inputs or uploads the patient’s data such as diagnosis and prognosis after hospital acceptance. All of these data, which comprise the smartphone app data, ambulance data, and hospital data, are merged in the ORION cloud server and managed as one large database in Osaka. To collect data from OMFD as well as emergency hospitals, we used a highly confidential line such as a virtual private network (VPN) rather than the Internet, and the server that could safely store massive data from these institutions was separated from the normal Internet line. In addition, we built up two backup servers in addition to the main server to avoid the loss of the ORION database. Analysis of the ORION data is fed back to every fire department and emergency hospital. Public health departments in Osaka will also be able to examine the effect of health policy on emergency medical system using these data (Figure 1).

This smartphone app of the ORION system was introduced in all areas of Osaka City at the same time on 1^st^ January, 2013 and has been working as of July 2017. In Osaka City, the other emergency medical system did not change during the study period except for the introduction of the ORION system.

### Data Collection and Quality Control

Data were uniformly collected using specific forms that included age, sex, foreigner, Glasgow Coma Scale (GCS), chronological factors such as the time of day and day of the week, the time course of transportation such as time of the call and hospital arrival, reason for the EMS call, and the total number of phone calls made to hospitals by EMS personnel at the scene. The data were completed by EMS personnel in cooperation with the physicians caring for the patient, transferred to the EMS Information Center of OMFD, and then checked by the investigators. If the dataset was incomplete, the investigators returned it to the responsible EMS personnel for completion of the data.

### Endpoint

The main endpoint was the difficulty in hospital acceptance. In this study, we defined the difficulty in hospital acceptance as the case in which EMS personnel at the scene needed to make ≥5 phone calls to medical institution before the hospital accepted the patient according to the guidelines regarding the transport and hospital acceptance of emergency patients in Osaka City [9].

### Statistical Analysis

As a primary analysis, we evaluated changes in the number of the difficulties in hospital acceptance for each month before and after the introduction of the smartphone app, with the use of interrupted time-series analysis to evaluate the introduction effect of a smartphone app on the difficulty in hospital acceptance [10]. In this study, we set the time point as January 2013, when this smartphone app was introduced and assessed the relationship between the number of difficulties in hospital acceptance by month and the elapsed time (months) since the study initiation, adjusting the month as a covariate to take seasonality into consideration. On the basis of previous studies [4,11], we also conducted subgroups such as age group (children <15 years, adults aged 15-64 years, and the elderly aged ≥65 years), sex (male or female), time of the day (daytime or nighttime), and day of the week (weekday or weekend or holiday). Furthermore, we assessed the introduction effect especially in emergency cases such as out-of-hospital cardiac arrest, traffic accident, injury by assault, and self-induced drug abuse or gas poisoning or trauma.

Patient and EMS characteristics between the two groups (<5 and ≥5 phone calls) were assessed by chi-square test for categorical variables and the Wilcoxon test for continuous variables. In this study, we defined emergency patients enrolled in the period from 2013 to 2015 after the introduction of the ORION system as the smartphone app group, that is, the group on which the smartphone app was used. As a sensitive analysis, we calculated the adjusted odds ratios (AORs) and 95% CIs with the use of a multivariable logistic regression model. We also considered potential confounding factors that existed before the EMS personnel made contact with the patient. These factors included age group (children <15 years, adults aged 15-64 years, and the elderly aged ≥65 years), sex (male or female), foreigner (yes or no), disturbance of consciousness (defined as GCS ≤8, or not), time of the day (daytime or nighttime), day of the week (weekday or weekend or holiday), seasonality (January-March, April-June, July-September, and October-December), use of the smartphone app (yes or no), and reason for the EMS call [1,12]. Reasons why a patient, the patient’s family, or bystanders called an ambulance included internal disease; gynecological disease; traffic accident involving vehicle, ship, or aircraft; industrial accident; sports-related disease and injury; asphyxia; trauma by assault; self-induced drug abuse or gas poisoning or trauma; other trauma; and others.

All tests were two-tailed, and *P* values of <.05 were considered statistically significant. Statistical analyses were performed using Statistical Package for the Social Sciences (SPSS) statistical package V.22.0J (IBM Corp).

## Results

### Study Population

**Figure 2:**
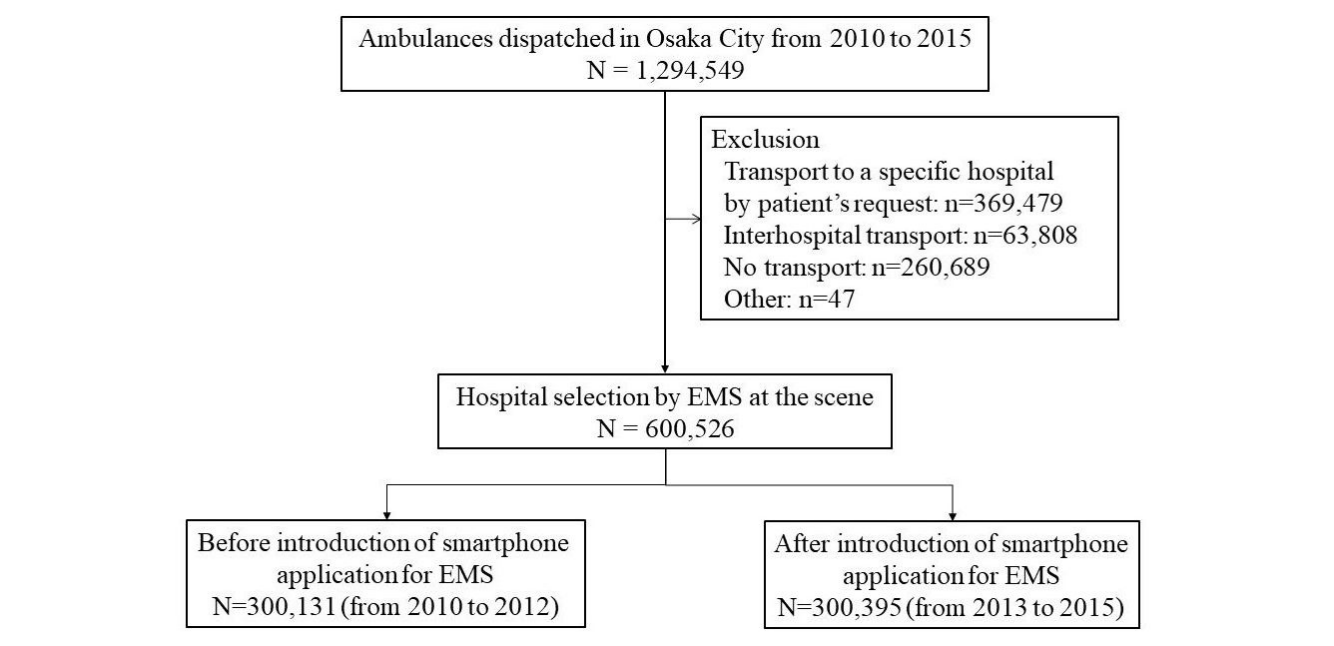
Patient flow during the study periods.

Figure 2 shows the flow of the enrolled patients during the study period from 2010 to 2015 in Osaka City. A total of 1,294,549 emergency dispatches were documented in Osaka City during the study period. A total of 600,526 emergency patients were included for our analysis (300,131 patients [50.00%] in the control group and 300,395 patients [50.00%] in the smartphone app group) after excluding 369,479 patients who were transported to the specific hospitals requested by patients or their family; 63,808 patients who underwent interhospital transport; 260,689 patients who were not transported by ambulance; and 47 patients with other reasons.

### Outcome

**Figure 3:**
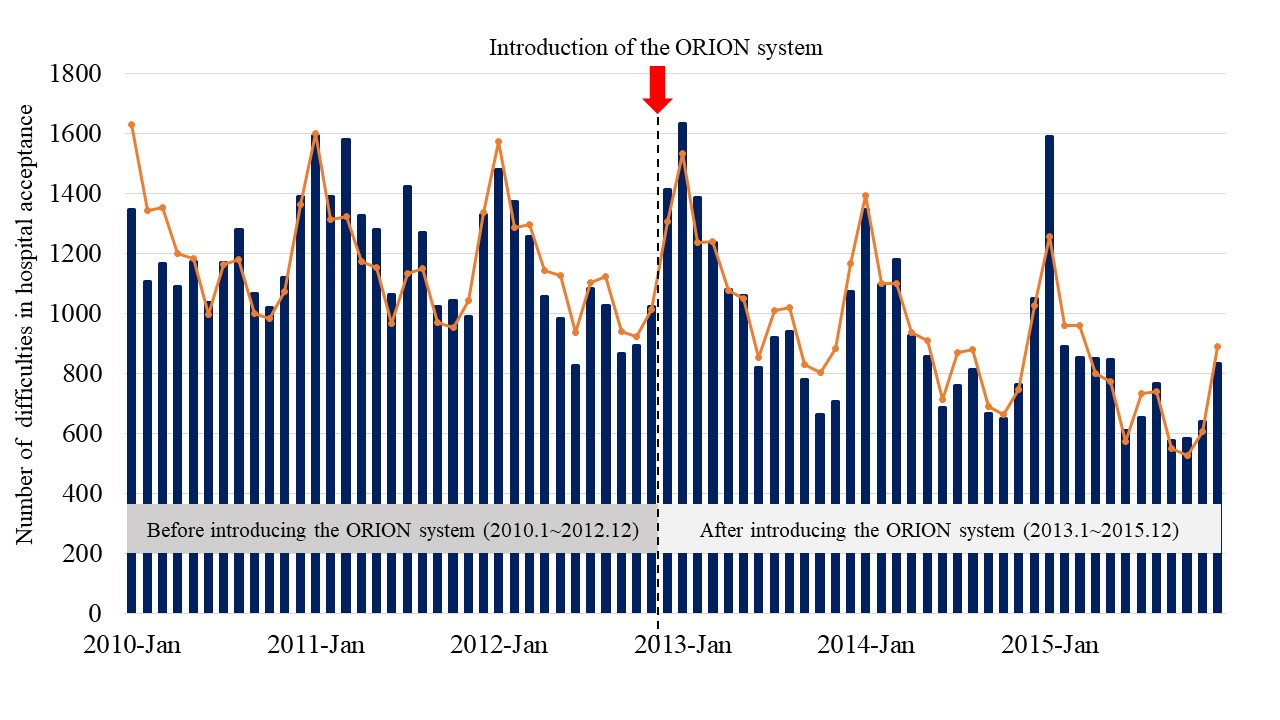
The number of difficulties experienced in hospital acceptance by month and the predicted number of difficulties in hospital acceptance by interrupted time-series analysis. The numbers of patients who had difficulty in hospital acceptance are shown by month with blue bars, and the predicted numbers of difficulties in hospital acceptance calculated from a regression formula with interrupted time-series design are shown by the orange line.

Figure 3 shows the number of patients who had difficulty in hospital acceptance (blue bars) by month and the predicted number of difficulties in hospital acceptance calculated from a regression formula with interrupted time-series design (orange line). The graph also shows that seasonality existed in the number of difficulties in hospital acceptance per month.

**Table 1:** Results of multiple linear regression analysis to detect association between the introduction of the smartphone app for the emergency medical service (EMS) system and the number of difficulties in hospital acceptance per month.

Object		Time trend before the introduction of the smartphone app (2010-2012) (change per month)			Time trend after the introduction of the smartphone app (2013-2015) (change per month)			Change in trends between pre- and postintervention period (2010-2015) (change per month)				*R*^2^
		Regression coefficient^a^	95% CI	*P* value	Regression coefficient^a^	95% CI	*P* value	Regression coefficient^a^	95% CI	*P* value		
All		−2.43	−5.49 to 0.64	.118	−11.61	−14.57 to −8.65	<.001	−9.18	−14.56 to −3.81	.001	.810	
**Subgroup**												
	Children	−0.67	−0.89 to −0.45	<.001	−0.54	−0.75 to −0.33	<.001	0.13	−0.25 to 0.52	.484	.723	
	Adult	−1.94	−3.62 to −0.25	.025	−7.00	−8.62 to −5.37	<.001	−5.06	−8.01 to −2.11	.001	.776	
	Elderly	0.18	−1.31 to 1.67	.807	−4.26	−6.87 to −1.65	.002	−4.08	−5.52 to −2.64	<.001	.839	
	Daytime	−0.95	−2.04 to 0.14	.087	−3.63	−4.69 to −2.57	<.001	−2.68	−4.59 to −0.77	.007	.801	
	Nighttime	−1.48	−3.65 to 0.69	.178	−7.99	−10.09 to −5.89	<.001	−6.51	−10.32 to −2.70	.001	.788	
	Weekday	−1.81	−3.67 to 0.06	.058	−6.94	−8.74 to −5.14	<.001	−5.13	−8.41 to −1.86	.003	.795	
	Weekend/Holiday	−0.62	−2.26 to 1.01	.450	−4.67	−6.25 to −3.09	<.001	−4.05	−6.92 to −1.19	.006	.774	
	Out-of-hospital cardiac arrest	0.01	−0.09 to 0.11	.827	−0.20	−0.30 to −0.11	<.001	−0.22	−0.39 to −0.04	.018	.791	
	Traffic accident	−0.26	−0.68 to 0.15	.205	−1.46	−1.85 to −1.06	<.001	−1.19	−1.91 to −0.47	.002	.617	
	Trauma by assault	−0.05	−0.26 to 0.15	.598	−0.34	−0.53 to −0.14	.001	−0.28	−0.64 to 0.07	.115	.346	
	Drug abuse, gas poisoning and trauma by self-injury	−0.44	−0.62 to −0.27	<.001	−0.40	−0.57 to −0.23	<.001	0.04	−0.26 to 0.35	.778	.663	

^a^Regression model was adjusted for seasonal effects.

No change over time in the number of difficulties in hospital acceptance was found before the introduction of the smartphone app (regression coefficient: −2.43, 95% CI −5.49 to 0.64), but after its introduction, the number of difficulties in hospital acceptance gradually decreased by month (regression coefficient: −11.61, 95% CI −14.57 to −8.65; Table 1). The regression coefficient of the changes in trends (slope change) between pre- and post-intervention periods was −9.18 (95% CI −14.56 to −3.81). In the subgroup analyses (Table 1), after the introduction of the smartphone app, the number of difficulties in hospital acceptance gradually decreased by month among adults (regression coefficient: −7.00, 95% CI −8.62 to −5.37) and the elderly (regression coefficient: −4.26, 95% CI −6.87 to −1.65). However, as for children, before its introduction, the number of difficulties in hospital acceptance gradually decreased (regression coefficient: −0.67, 95% CI −0.89 to −0.45), and the introduction effect before and after its introduction was not observed in this group (regression coefficient: 0.13, 95% CI −0.25 to −0.52). In all chorological groups, the number of difficulties in hospital acceptance gradually decreased after the introduction of the smartphone app. As for emergency cases, after the introduction of the smartphone app, the number of difficulties in hospital acceptance gradually decreased by month in out-of-hospital cardiac arrest (regression coefficient: −0.20, 95% CI −0.30 to −0.11) and traffic accident (regression coefficient: −1.46, 95% CI −1.85 to −1.06). However, no change over time in the number of difficulties in hospital acceptance was found before and after the introduction of the smartphone app in trauma by assault (regression coefficient: −0.28, 95% CI −0.64 to 0.07) and drug abuse or gas poisoning or trauma by self-injury (regression coefficient: 0.04, 95% CI −0.26 to 0.35).

Patient and EMS characteristics before and after the introduction of the smartphone app are shown in Table 2. Patients in the smartphone app group were more likely to be older and female, have a lower proportion of disturbance of consciousness, and have a higher proportion of occurrence on weekend and holiday compared with the control group. The number of foreigners treated after the introduction of the smartphone app was higher than that before its introduction. Although the time interval from patient call to contact was similar between the two groups, the time interval from patient call to hospital arrival in the smartphone app group was longer than that of the control group.

**Table 2:** Patient characteristics before and after the introduction of the smartphone app for emergency medical service (EMS).

Characteristics		Before the introduction of the smartphone app for EMS^a^ (2010-2012) n=300,131		After the introduction of the smartphone app for EMS (2013-2015) n=300,395		*P* value	
Age, median (IQR^b^)		49 (24-74)		50 (25-75)		<.001	
**Age group in years, n (%)**						<.001	
	Children aged ≤14 years		27,892 (9.29)		26,656 (8.87)		
	Adults aged 15-64 years		171,316 (57.08)		164,959 (54.91)		
	Elderly aged ≥65 years		100,923 (33.63)		108,778 (36.21)		
Male, n (%)		168,559 (56.16)		164,826 (54.87)		<.001	
Foreigner, n (%)		542 (0.18)		1227 (0.41)		<.001	
Disturbance of consciousness (GCS^c^≤8), n (%)		16,721 (5.57)		16,331 (5.44)		.026	
**Time of day, n (%)**						.897	
	Daytime (9:00 am-5:00 pm)		125,885 (41.94)		126,071 (41.97)		
	Nighttime (5:00 pm-9:00 am)		174,246 (58.06)		174,322 (58.03)		
**Day of week, n (%)**						<.001	
	Weekday		190,796 (63.57)		188,838 (62.86)		
	Weekend or holiday		109,335 (36.43)		111,555 (37.14)		
**Seasonality, n (%)**						<.001	
	January-March		73,534 (24.50)		75,573 (25.16)		
	April-June		72,148 (24.04)		72,339 (24.08)		
	July-September		78,701 (26.22)		77,211 (25.70)		
	October-December		75,748 (25.24)		75,271 (25.06)		
**Reason for EMS call, n (%)**						<.001	
	Internal disease		185,196 (61.71)		180,097 (59.95)		
	Gynecological disease		3040 (1.01)		3190 (1.06)		
	Traffic accident by car, ship, or aircraft		41,834 (13.94)		38,438 (12.80)		
	Injury, toxication, and disease by industrial accident		3373 (1.12)		3756 (1.25)		
	Sports-related disease and injury		2362 (0.79)		2533 (0.84)		
	Asphyxia		1315 (0.44)		1421 (0.47)		
	Trauma by assault		51,480 (17.15)		53,662 (17.86)		
	Drug abuse, gas poisoning, and trauma by self-injury		6047 (2.01)		5560 (1.85)		
	Other injury		4806 (1.60)		4149 (1.38)		
	Others		678 (0.23)		587 (0.20)		
Time from patient’s call to contact by EMS in minutes, median (IQR)		5 (3-6)		5 (3-6)		<.001	
Time from patient’s call to hospital arrival in minutes, median (IQR)		29 (23-39)		31 (24-41)		<.001	

^a^EMS: emergency medical service.

^b^IQR: interquartile range.

^c^GCS: Glasgow Coma Scale.

**Table 3:** Number of phone calls and time interval for hospital selection before and after the introduction of the smartphone app for emergency medical service (EMS).

Outcome	Before the introduction of the smartphone app for EMS^a^ (2010-2012) n=300,131	After the introduction of the smartphone app for EMS (2013-2015) n=300,395	*P* value
Number of phone calls until hospital acceptance, median (IQR^b^)	2 (1-3)	1 (1-3)	<.001
Time interval of hospital selection by EMS at the scene in minutes, median (IQR)	4 (2-10)	4 (3-9)	.012
Number of cases needing only one call by EMS until hospital acceptance, n (%)	143,050 (47.66)	154,987 (51.59)	<.001
Number of cases needing ≥5 calls by EMS until hospital acceptance, n (%)	42,585 (14.19)	32,819 (10.93)	<.001
Time interval from EMS scene arrival to hospital arrival in minutes, median (IQR)	24 (16-32)	26 (18-34)	<.001

^a^EMS: emergency medical service.

^b^IQR: interquartile range.

**Table 4:** Sensitivity analysis of ≥5 calls to hospitals by on-scene emergency medical service (EMS) personnel before and after the introduction of the smartphone app by using a multivariable logistic regression analysis.

Outcome		Percentage of difficulty in hospital acceptance % (n/N)	OR^a^ adjusted	95% CI	*P* value
**Introduction of a smartphone app for EMS^b^**					
	Before the introduction of a smartphone app	14.19 (42,585/300,131)	Reference		
	After the introduction of a smartphone app	10.93 (32,819/300,395)	0.73	0.72-0.74	<.001

^a^OR: odds ratio.

^b^EMS: emergency medical service.

Table 3 shows the number of phone calls and the time taken for hospital selection by EMS personnel with or without the smartphone app. The hospital selection time by EMS personnel until hospital acceptance was similar between the two groups. However, the median number of phone calls was lower in the smartphone app group than in the control group (1 [IQR: 1-3] vs 2 [IQR: 1-3] calls, *P*<.001). The proportion of emergency patients who were accepted by the first hospital called was higher in the smartphone app group than in the control group (51.59% vs 47.66%, *P*<.001), and the proportion of those requiring ≥5 phone calls until hospital acceptance was lower in the smartphone app group than in the control group (10.93% [32,819/300,395] vs 14.19% [42,585/300,131], *P*<.001). The time interval from EMS scene arrival to hospital arrival was significantly longer in the smartphone app group than that in the control group (26 [IQR: 18-34] vs 24 [IQR: 16-32] min, *P*<.001).

The results from a multivariable logistic regression analysis assessing the effects of the introduction of the smartphone app are shown in Table 4. The AOR for the difficulty in hospital acceptance before and after the introduction of the smartphone app was 0.73 (95% CI 0.72-0.74).

## Discussion

### Principal Findings

From the population-based ambulance records in Osaka City, Japan, we evaluated the changes in the number of difficulties in hospital acceptance by month before and after the introduction of the smartphone app with the use of interrupted time-series analysis. Although there were no significant changes in the number of difficulties in hospital acceptance before the introduction of the smartphone app, the number of difficulties in hospital acceptance after the introduction of the smartphone app gradually decreased over time. Therefore, considering our results that the number of difficulties in hospital acceptance gradually decreased by month after the introduction of the smartphone app, we believe that a change in health policy, such as the introduction of a smartphone app, appeared to gradually affect the practice on the front line after the app’s introduction. In other words, it appeared to take time for the on-scene EMS personnel to make full use of this app.

Furthermore, we revealed that the introduction of the smartphone app for the EMS system in prehospital settings reduced the difficulty in obtaining hospital acceptance. The ORION system was comprehensively introduced and is operated in both emergency medical institutions and the municipal fire department in Osaka City, one of the biggest cities in Japan. When EMS personnel select an appropriate hospital for emergency patients, this app enables them to share both the real-time information on the transport situation of other ambulances and the treatment status of other patients after transport. Considering this information, EMS personnel can transport emergency patients to the selected hospital according to patient severity, and the introduction of this app has led to a decrease in the difficulty in obtaining hospital acceptance in this area. Our findings showing improvement of the EMS system by the introduction of an IT system also reinforce the importance of IT in prehospital settings.

On the other hand, the time interval from EMS scene arrival to hospital arrival in this study was significantly longer in the smartphone app group than that in the control group. Although this study defined the difficulty in hospital acceptance as emergency cases required ≥5 phone calls to a medical institution before the hospital accepted the patient according to the guidelines in Osaka City [9], this result suggests that there might be emergency cases with longer time from EMS scene arrival to hospital arrival with less phone calls in the smartphone app group. However, we consider that it is not appropriate to evaluate the improvements of difficulty in hospital acceptance only by the time interval. For example, it would be important that EMS personnel at the scene are able to transport emergency patients with less phone calls to the distant appropriate hospital that can treat them by advanced procedures, even if the hospital arrival time prolonged. Therefore, in the future, data collection and analysis about treatments after hospital arrival, as well as the time interval from an EMS arrival to in-hospital treatments is also needed to evaluate the improvement effect of difficulty in hospital acceptance by this smartphone app.

Several previous studies have demonstrated that sharing information on the transport situation between medical institutions and ambulances can lead to improvement of an EMS system. McLeod and colleagues [13] reported that a medical information system that shared information about hospital capacity according to the severity of emergency patients reduced ambulance avoidance and improved patient outcome in Calgary City, Canada. Raaber and colleagues [14] demonstrated that obtaining information about the ambulance situation in the emergency department with the use of a geographic information system was associated with a reduction in the waiting time of the trauma team and medical emergency team and improvement in the nurses’ workflow when each hospital received emergency patients in Horsens City, Central Denmark. The population density of Osaka City is approximately 12,000 people/km^2^ and is much higher than that of Calgary City (1360 people/km^2^) and Horsens City (159 people/km^2^) [15-17], and as such, the 99 emergency medical institutions, including 6 critical care centers in this area, must receive over 220,000 emergency patients transported by ambulances every year [12]. Therefore, the EMS system of Osaka City is always congested, and the request for patient transport by EMS is more likely to exceed the capacity of hospital acceptance. Therefore, sharing real-time information between medical institutions and ambulances with the smartphone app was of help in conducting hospital selection more appropriately by EMS personnel at the scene in Osaka City. From the viewpoint of this and other previous studies, regardless of the size of the city or the difference in EMS systems, the introduction of IT in prehospital settings would appear to contribute to facilitating the EMS system, including hospital acceptance or treatment after hospital arrival.

In addition, some studies also showed improvements in ambulance diversion with the use of the Internet. Lagoe and colleagues [18] reported on an Internet system monitoring the number of ambulance diversions and interhospital transports in Syracuse, New York, but this system updates the situation only once a day. Sprivulis and colleagues [19] revealed that sharing information about the situation of patient acceptance in each emergency department via the Internet improved ambulance diversion in Perth, Western Australia. This system enabled emergency physicians and nurses to share information about each emergency department at 5-min intervals via a patient tracking system, but the method of tracking patients was not illustrated in their paper. In our smartphone app, when EMS personnel record the time course, such as the time of arrival at the scene or hospital arrival, the ambulance status on the app also changes, and this information is shared on the ORION cloud server, and the status of other ambulances is synchronously updated on the smartphone app. By utilizing this function, EMS personnel can be simply apprised of information on patient transport and hospital acceptance in real time without bothering the hospital staff.

In children, the difficulty in hospital acceptance improved both before and after the introduction of the smartphone app, but change in trend between pre- and post-intervention period was not recognized in a subgroup analysis. As shown in our previous study, emergency medical system for pediatric patients has been well worked before its introduction [4], and the use of a smartphone app for children was not associated with difficulty in hospital acceptance in Osaka City. In cases with trauma by assault and self-injury, no changes in trend were also observed by the introduction of a smartphone app. Although the cooperation between the emergency department and the psychiatry department is necessary to accept self-injured emergency patients, its cooperative relationship has not been sufficiently built up in Osaka City. Therefore, the smartphone app as a tool to search for an appropriate hospital with both departments might not be effective. In Japan, police officers are rarely stationed in medical institutions, and cooperation between the police and medical institutions is not sufficiently established. Therefore, there might be few medical institutions that accept patients with crime-related injuries in Japan, even if EMS personnel selected an appropriate hospital for such patients with the smartphone app. Thus, the improvement effect on the difficulty in hospital acceptance by the introduction of a smartphone app differed in some subgroups because of various factors. Both IT and efforts to improve the patient acceptance system are needed to comprehensively improve the EMS system and further reduce the difficulty in hospital acceptance in the future.

### Limitations

This study has some inherent limitations. First, the purpose of this study was to assess whether the introduction of a smartphone app reduced the difficulty in hospital acceptance, and we did not assess the effect on the prognosis of the emergency patients. The ORION system has been collecting in-hospital data including patient prognosis since 2015, and we will assess this aspect in the future. Second, we assessed the effect of the smartphone app’s introduction based on the unified definition of the difficulty in hospital acceptance regardless of pathological condition, but it may be necessary to define and assess disease-specific difficulty in hospital acceptance. For example, the time interval from onset to call to the start of percutaneous coronary intervention for acute coronary syndrome is one example of an important index [20,21]. However, we could not obtain such information before the introduction of the smartphone app during the study period. Third, we did not have information about potential factors that could affect the improvements of difficulty in hospital acceptance, such as the adherence of the ORION use (ie, the actual rate of using a smartphone app), the decision making of on-scene EMS personnel to select a hospital from the hospital list by this app, and the number of emergency departments (EDs), ED beds, and ED providers before and after intervention. Finally, this study was an observational study, and there may be unknown confounding factors associated with the difficulty in hospital acceptance.

### Conclusions

We developed a smartphone app for the EMS system that enables EMS personnel at the scene to share various information regarding patient transport by other ambulances or treatment of patients in medical institutions in Osaka City, Japan. Sharing of such information between the ambulances and hospitals in the prehospital setting was associated with decreasing difficulty in hospital acceptance. Our findings may be considered useful for developing an emergency medical information system using IT in other areas of the world.

## Appendix

Multimedia Appendix 1 ORION smartphone app screenshot for time records. When EMS personnel launch this app and register an emergency patient, the app screen for recording the prehospital time course regarding patient transport is active.

Multimedia Appendix 2 ORION smartphone app screenshot for patient status. When EMS personnel at the scene touch the button “To patient check list” in the ambulance status screen, the app screen for recording patient status, such as vital signs and background, is active.

Multimedia Appendix 3 ORION smartphone app screenshot for assessing a patient with chest pain. EMS personnel can choose symptoms displayed on the app screen that match the patient’s complaints, and then the appropriate hospitals are listed based on the patient’s condition.

Multimedia Appendix 4 ORION smartphone app screenshot for the hospital list. When EMS personnel select the check items on the screen, the app shows the list of hospitals that can conduct necessary treatments.

## Abbreviations

AOR: adjusted odds ratio
EMS: emergency medical service
GCS: Glasgow Coma Scale
IQR: interquartile range
IT: information technology
OMFD: Osaka Municipal Fire Department
OR: odds ratio
ORION: Osaka emergency information Research Intelligent Operation Network system
SPSS: Statistical Package for the Social Sciences
VPN: virtual private network
XML: Extensible Markup Language
