# Supplementary figures and images for: Improvements in Patient Acceptance by Hospitals Following the Introduction of a Smartphone App for the Emergency Medical Service System: A Population-Based Before-and-After Observational Study in Osaka City, Japan
Source: JMIR Mhealth Uhealth. 2017 Sep 11;5(9):e134. doi: 10.2196/mhealth.8296 (PMC5616023; doi:10.2196/mhealth.8296)

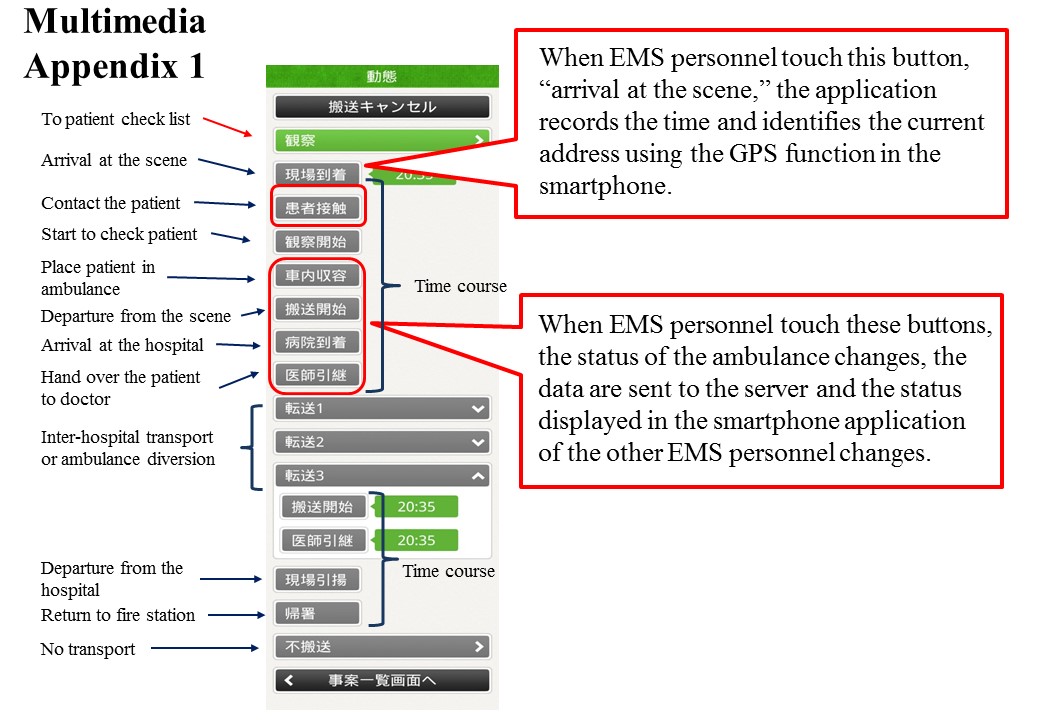

Supplement: Multimedia Appendix 1 [file mhealth_v5i9e134_app1.jpg]

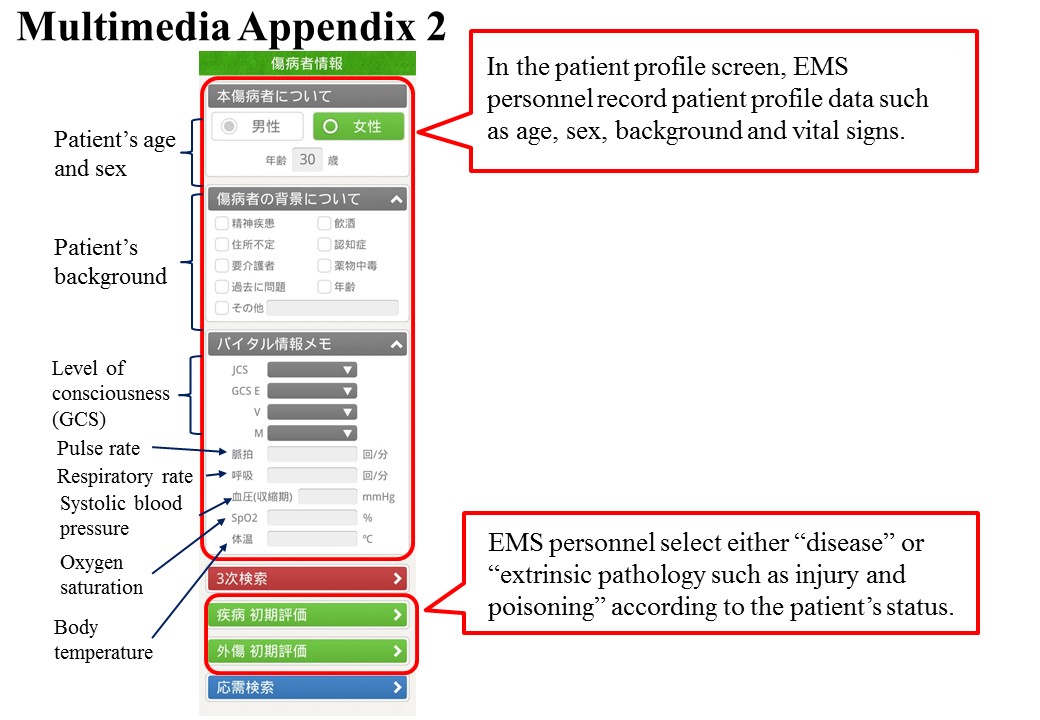

Supplement: Multimedia Appendix 2 [file mhealth_v5i9e134_app2.jpg]

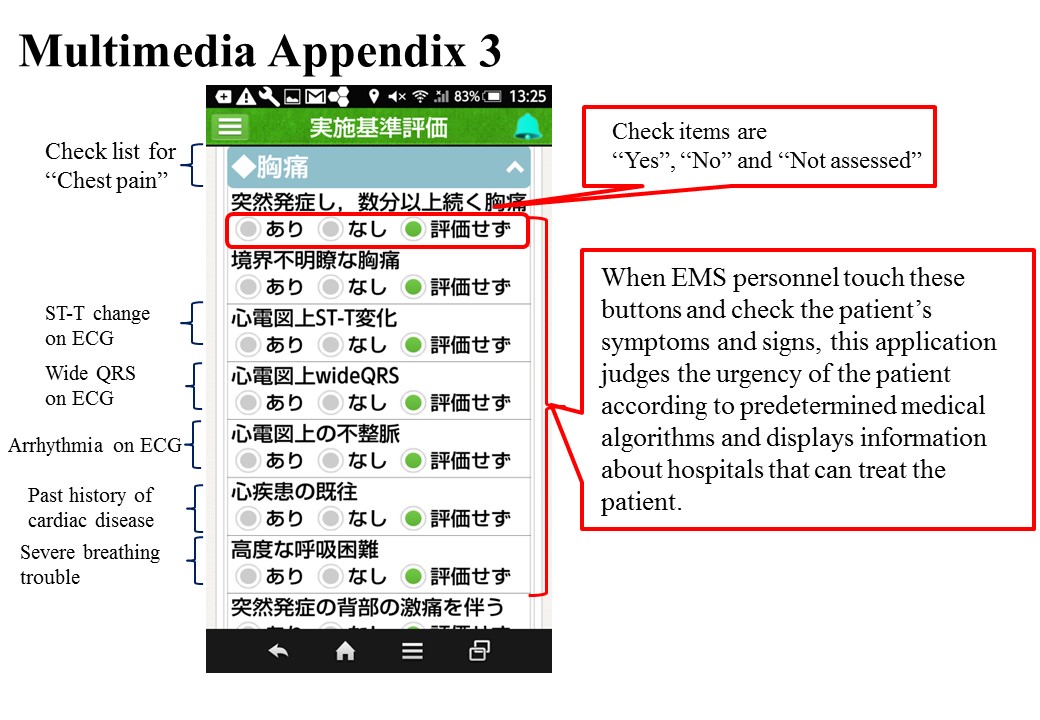

Supplement: Multimedia Appendix 3 [file mhealth_v5i9e134_app3.jpg]

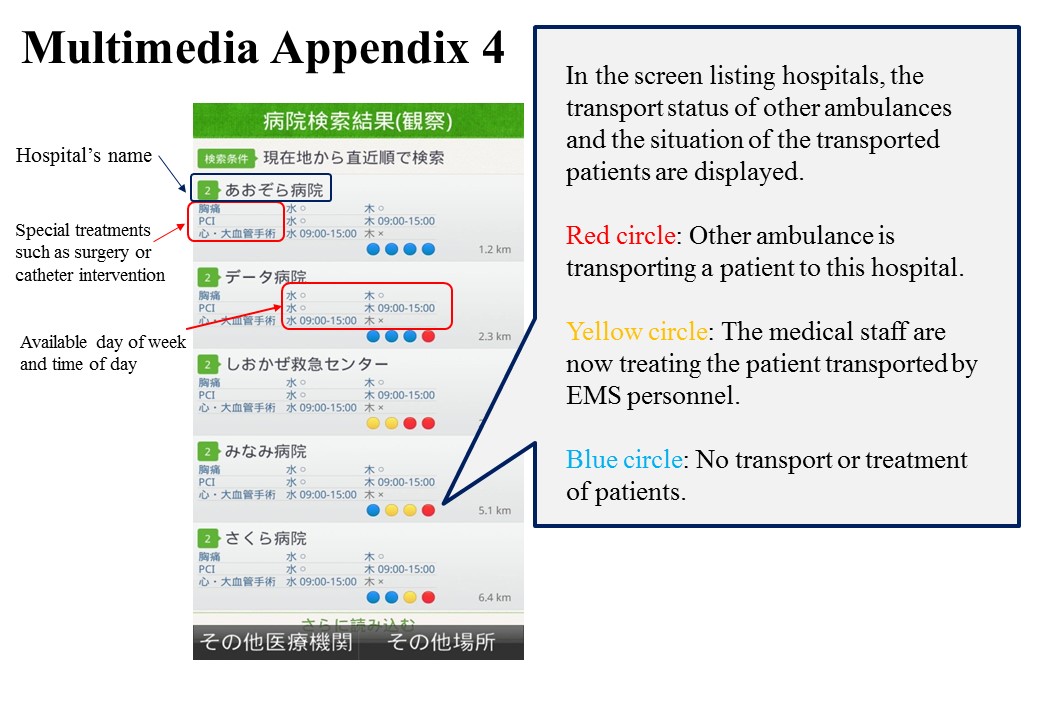

Supplement: Multimedia Appendix 4 [file mhealth_v5i9e134_app4.jpg]
